# Flies and Their Breeding Places

**Published:** 1910-04-02

**Authors:** 


### FLIES AND THEIR BREEDING PLACES.

Owing to the fact that the infection of milk, for instance, by bacteria carried by the feet of flies may be no inconsiderable factor in the problem of summer diarrhœa in infants, and owing to the possibility that flies may be a similar potent factor in the spread of other bacterial infections, such as typhoid fever, colds, and so forth, it becomes important as a prophylactic measure in limiting the number of flies that may be present in a given neighbourhood to know something about their breeding places in the first instance.

The chief breeding places of the house-fly are : (*a*) Stable middens containing fermenting manure or a mixture of this and cow-dung; (*b*) middens containing fermenting spent hops; and (*c*) ashpits containing fermenting vegetable matter—that is to say, about 25 per cent. of the total number of pits examined.

The conclusions arrived at as the result of extensive investigations upon the subject are :—

1. That covered ashpits and middens are as badly infested as those that are open.
2. That house-flies breed in all temporary collections of fermenting matter.
3. That house-flies breed in relatively small numbers in ashpits where no fermentation takes place.
4. That they do not breed in ashpits that are emptied at short intervals or in the patent bins.
5. That the use of disinfectants in ashpits does not absolutely prevent the flies breeding in such receptacles.
6. That very dry or excessively wet ashes or moist cow-dung does not harbour them.
7. That the presence of fowls (not ducks or geese), having free access to the stable middens, reduces the number of larvæ and pupæ to a very marked extent.
8. That the life-cycle of the fly, in all kinds of fermenting materials, is reduced to the minimum period of ten to fourteen days; and that in the absence of such artificial heat the cycle may occupy a period of from three to five weeks or more.
9. That house-flies do not depend entirely upon excessively warm weather for breeding purposes, though in hot seasons they breed much more rapidly in non-fermenting materials, and their numbers, under such conditions, become greatly increased.

If house-flies are to be reduced to a minimum, therefore, the following suggestions might be adopted :—

1. Stable manure and spent hops should not be allowed to accumulate in the middensteads during the months of May to October inclusive for a period of more than seven days.
2. All middensteads should be thoroughly emptied and carefully swept at least once a week. The present system of partly emptying such receptacles should in all cases be discontinued. The walls of middensteads should also be cemented over, or, failing this, the brickwork should be sound and well pointed.
3. All ashpits should be emptied during the summer months at intervals of not more than ten days.
4. The most strenuous efforts should be made to prevent children defæcating in courts and passages, or the parents should be compelled to remove such matter immediately; and defæcation in stable middens should be strictly forbidden. The danger lies in the overwhelming attraction which such fæcal matter has for house-flies, which latter may afterwards come into direct contact with man or his foodstuffs.
5. Ashpit refuse which in any way tends to fermentation, such as bedding, straw, old rags, paper, waste vegetables, dirty bedding from the "hutches" of pet animals, etc., should if possible be disposed of by tenants, preferably by incineration, or be placed in a separate receptacle so that no fermentation could take place. If such precautions were adopted by householders relatively few house-flies would breed in the ashpits, and the present system of emptying such places at longer intervals than, say, four to six weeks might be continued.
6. The application of Paris green (poison) at the rate of 2 oz. to one gallon of water to either stable manure or ashpit refuse will destroy 99 per cent. of the larvæ. Possibly a smaller percentage of Paris green might be employed with equally good results. One per cent. of crude atoxyl in water kills 100 per cent. of fly larvæ. The application of either of these substances might, however, lead to serious complications, and it is very doubtful whether they could be employed with safety. Paris green at a rate of 1 oz. to 2 oz. to twenty gallons of water is used largely as an insecticide for fruit pests. It does no harm to vegetation when applied in small quantities; but cattle might possibly eat the dirty vegetable matter that had been treated with this substance, and the results might prove fatal if large quantities were eaten.
7. The use of sun-blinds in all shops containing food which attracts flies would largely reduce the number of flies in such places during hot weather. Small fruiterers' and confectioners' shops, as a rule, are not shaded by sun-blinds, and in their absence flies literally swarm on the articles exposed for sale.
8. The screening of middensteads with fine wire gauze would undoubtedly prevent flies from gaining access to manure, etc., but it is very doubtful if this method would meet with any marked success. The gauze would rapidly oxidise, the framework supporting it would probably warp, and numbers of flies would be admitted whenever the receptacle was opened. Moreover, the erection of such a structure would prove a great inconvenience and a hindrance to the removal of the refuse. This, however, does not prejudice the possibility of inventing a good fly-proof screen.

